# Comparative genome analyses of Staphylococcus aureus from platelet concentrates reveal rearrangements involving loss of type VII secretion genes

**DOI:** 10.1099/acmi.0.000820.v4

**Published:** 2024-09-13

**Authors:** Sylvia Ighem Chi, Annika Flint, Kelly Weedmark, Franco Pagotto, Sandra Ramirez-Arcos

**Affiliations:** 1 Medical Affairs and Innovation, Canadian Blood Services, Ottawa, Ontario, Canada; 2 Department of Biochemistry, Microbiology and Immunology, University of Ottawa, Ottawa, Canada; 3 Listeriosis Reference Centre, Microbiology Research Division, Bureau of Microbial Hazards, Food Directorate, Health Canada, Ottawa, Canada; 4 Microbiology Research Division, Bureau of Microbial Hazards, Food Directorate, Health Canada, Ottawa, Canada

**Keywords:** comparative genomics, rearrangements, *S. aureus* genomes, type VII secretion system, virulence

## Abstract

*Staphylococcus aureus* has been involved in transfusion-transmitted fatalities associated with platelet concentrates (PCs) due to its heightened pathogenicity enhanced by genome-encoded virulence and antibiotic resistance genes. This may be facilitated by mobile genetic elements (MGEs) that can cause rearrangements. Several factors contribute to *S. aureus* virulence, including the type VII secretion system (T7SS), composed of six core genes conserved across *S. aureus* strains. In this study, we conducted comparative genome analyses of five *S. aureus* isolates from PCs (CI/BAC/25/13 /W, PS/BAC/169/17 /W and PS/BAC/317/16 /W were detected during PCs screening with the BACT/ALERT automated culture system, and ATR-20003 and CBS2016-05 were missed during screening and caused septic transfusion reactions). Multiple alignments of the genomes revealed evidence of rearrangements involving phage Sa3int in PS/BAC/169/17 /W and PS/BAC/317/16 /W. While the former had undergone translocation of its immune evasion cluster (IEC), the latter had lost part of the phage, leaving behind the IEC. This observation highlights *S. aureus* genome plasticity. Unexpectedly, strain CBS2016-05 was found to encode a pseudo-type VII secretion system (T7SS) that had lost five of the conserved core genes (*esxA*, *esaA, essA, esaB* and *essB*) and contained a 5′ truncated *essC*. Since these genes are essential for the function of the T7SS protein transport machinery, which plays a key role in *S. aureus* virulence, CBS2016-05 probably compensates by recruiting other export mechanisms and/or alternative virulence factors, such as neu-tralizing immunity proteins. This study unravels genome rearrangements in *S. aureus* isolated from PCs and reports the first *S. aureus* isolate lacking conserved T7SS core genes.

## Data Summary

The complete *de novo* genome sequences of the newly sequenced *S. aureus* strain ATR-20003 and previously sequenced strains CBS2016-05 (CBS), CI/BAC/25/13 /W (CI/25), PS/BAC/169/17 /W (PS/169) and PS/BAC/317/16 /W (PS/317) used in this study are available at GenBank at the following corresponding sites: Staphylococcus aureus strain ATR-20003chromosome, complete genome - Nucleotide - NCBI (nih.gov), Staphylococcus aureus strain CBS2016-05 chromosome, complete genome - Nucleotide - NCBI (nih.gov), Staphylococcus aureus strain CI/BAC/25/13 /Wchromosome, complete genome - Nucleotide - NCBI (nih.gov), Staphylococcus aureus strain PS/BAC/169/17 /Wchromosome, complete genome - Nucleotide - NCBI (nih.gov), Staphylococcus aureus strain PS/BAC/317/16 /Wchromosome, complete genome - Nucleotide - NCBI (nih.gov), with their respective BioProject ID numbers: PRJNA799255, PRJNA703931, PRJNA703973, PRJNA703966, and PRJNA703976. Supplementary files can be found in Figshare: https://doi.org/10.6084/m9.figshare.25958587.v1[1].

**Impact Statement**

Platelet concentrates (PCs) are a life-saving blood product used to treat patients with bleeding disorders and require stringent quality control which involves screening for microbial contamination or treatment with pathogen reduction technologies. Although implementation of PC screening with automated culture methods is a gold standard practice that has significantly improved the safety of this blood product, some bacteria rarely escape detection and can cause septic transfusion reactions in susceptible recipients. The clinical outcome of patients receiving contaminated PCs depends on the bacterial load of the transfused product, the virulence of the contaminant organism and the immune status of the recipient. This study focuses on the comparative genomic analysis of five *S. aureus* strains isolated from contaminated PCs, including two strains that escaped detection during PC screening and caused transfusion reactions. *S. aureus* is a common PC contaminant and is considered a major threat to patients due to its large repertoire of virulence factors including antibiotic resistance, production of exotoxins and biofilm formation. Our genomic analysis describes rearrangements involving phage Sa3int, which enhances its virulence potential. The most important finding reported herein is the discovery of a truncated type VII secretion system gene operon in the CBS2016-05 strain. The type VII secretion system is highly conserved within *S. aureus* isolates, has a key role in the secretion of virulence factors, and contributes to maintaining cell membrane integrity as well as interbacterial competition. It was therefore surprising to find that a highly virulent strain, which was involved in a septic transfusion event, lacks this important secretion system. Our findings indicate that virulent *S. aureus* strains may have other mechanisms to fulfil the functions of the type VII secretion system, which merits further investigation.

## Introduction

*Staphylococcus aureus* is a Gram-positive component of the human microbiota, but as an opportunistic pathogen it can cause diverse community and hospital-acquired infections, including life-threating bacteremia, necrotizing pneumonia, and endocarditis [2,3]. This pathogen can colonize various ecological niches within its human host, including the respiratory tract, skin, and nasal passages [4,5]. In medical settings, invasive *S. aureus* infections and bacteremia can result from *S. aureus* accessing the intravascular space via prosthetic devices such as surgical implants and central venous catheters [6,7]. In the domain of transfusion medicine, *S. aureus* residing on the skin can contaminate blood products during blood collection, where a portion of the skin at the venipuncture area passes through the injected needle into the collected blood [8]. *S. aureus* contamination of transfusable blood products, including platelet concentrates (PCs), has been reported in the USA, Europe, Canada and Africa [9,13], where sepsis, fatalities and death continue to pose serious safety concerns.

To understand the pathogenicity underlying *S. aureus* success in invading its host, manipulating the immune system, causing infections, and becoming resistant to treatment with antibiotics, researchers have sequenced and analysed complete genomes of *S. aureus*. These genomic DNAs have been isolated from diverse human samples, including PCs, whole blood, and non-blood sources like skin, nose, and trachea [14,28]. *S. aureus* remains a healthcare burden due to its constant evolving virulence and antimicrobial resistance (AMR) portfolios. This bacterium produces a plethora of virulence factors that facilitate attachment, colonization, cell-to-cell interactions, immune evasion, and tissue damage [29,30]. These include adhesins, exoenzymes, exotoxins, immune modulators, and an effector delivery type VII secretion system (T7SS). There does not appear to be a distinction between commensal and pathogenic strains regarding virulence genes.

The *S. aureus* T7SS operon comprises genes encoding for four core membrane proteins (EsaA, EssA, EssB, EssC) [31], and a subtstrate cluster made of EsaD, a nuclease toxin [32], EsaE, a chaperone [33], secretory proteins EsxACBD [34], a cytosolic regulator EsaB, and an immunity protein, EsaG [32]. Only the EssC1 variants of the T7SS have the substrate cluster [35], and many paralogue copies of EsaG and other orphan T7SS associated immunity genes are located downstream of *esaG* [36]. Six of these genes (*esxA, esaA, essA, esaB, essB* and *essC*), essential for protein transport, form the core component of T7SS and are conserved across *S. aureus* strains [31,37]. EssC is a multidomain membrane-bound ATPase that mediates substrate recognition and protein transport [37]. T7SS plays an important role in *S. aureus* virulence, ensuring survival and persistence [31,37, 38]. The virulence effect on the infected host is further complicated as treatment of *S. aureus* infections with antibiotics is becoming more challenging with the emergence of multidrug-resistant *S. aureus* strains (MDRSA) [39,41]. Moreover, vancomycin-resistant *S. aureus* strains have compounded the problem as they are often resistant to other commonly used antibiotics [42]. Several comparative genome studies revealed a diverse panoply of AMR including efflux pumps and target modifications that confer resistance to methicillin/penicillin, fluoroquinolones, fosfomycin, and aminoglycosides, among others [41,43, 44].

The horizontal transmission of genes that confer virulence and antibiotic resistance genes between bacteria is mediated by mobile genetic elements (MGEs) [45,46]. *S. aureus* genomes contain approximately 15–20% MGEs of the total genome size, which ranges from 2.5 to 2.9 Mbp with a typical average of 2.8 Mbp [47] and can influence the bacterial phenotype and genotype. The MGEs of *S. aureus* comprise a complex mix of *S. aureus* genomic islands (νSa), prophages (φSaint), plasmids, transposons, and staphylococcal chromosome cassettes (SCC) [48,49]. *S. aureus* harbours three main pathogenicity islands (νSaα, νSaβ and νSaγ) and four phage types (ɸSa1int to ɸSa4int) [45,50]. The hallmark features of νSaα are lipoprotein and superantigen-like gene clusters, while νSaβ is characterized by clusters of serine proteases and enterotoxin genes. Genes *ssl11* to *ssl14, hla, efb, ecb,* and *scn/sbi* are typical components of νSaγ [51]. Among the bacteriophages identified in *S. aureus* genomes, ɸSa3int is the most prevalent, typically carrying the bi-component leucocidin *lukHG* and immune evasion cluster (IEC) of *chp, sak, scn* and *eap* [52].

Whole genome studies of clinical *S. aureus* strains isolated from various sources can provide insights on virulence, antimicrobial resistance, and the acquisition of MGEs. Still, genomes of *S. aureus* from PCs, have not been examined in detail. In this study we performed a comprehensive genome analysis of five *S. aureus* isolates from PCs and twenty isolates from diverse sources (ten isolated from blood infections and ten non-blood isolates), with specific attention to genes encoding for virulence, antimicrobial resistance, and MGEs, and compared those results to determine if there was a correlation between isolate origin and specific genes.

## Methods

### *S. aureus* isolates

The complete genome of the newly sequenced *S. aureus* ATR-20003 (ATR), isolated from a PC unit involved in a septic transfusion event [11], was used in this study, together with the whole genomes of four other PC strains recently reported [CBS2016-05 (CBS), CI/BAC/25/13 /W (CI/25), PS/BAC/169/17 /W (PS/169) and PS/BAC/317/16 /W (PS/317)] [14,17]. To investigate the genomic characteristics of the five *S*. *aureus* strains isolated from PCs, their closed genomes were compared to 20 clinical *S. aureus* strains publicly available in the NCBI database that originated from human specimens. Therefore, a total of 25 *S*. *aureus* genomes were included in this study: five PC isolates, ten blood isolates involved in infection [18,21], and ten non-blood strains [22,28] (Table 1), representing a small portion (~1%) of available *S. aureus* genomes.

**Table 1.** Overview of *S. aureus* genome features from PC isolates in comparison to other blood and non-blood strains

*S. aureus* strain	Source	Chromosome size (bp)	GC content (%)	CDSs	rRNAs	tRNAs	ncRNAs	Accession #	Reference
ATR-20003	Blood (PC)	2 764 272	32.94	2 630	19	58	4	CP091412	This study
CBS2016-05	Blood (PC)	2 766 936	32.87	2 740	19	59	4	CP070991	[14]
CI/BAC/25/13 /W	Blood (PC)	2 719 347	32.88	2 629	19	60	4	CP071102	[15]
PS/BAC/169/17 /W	Blood (PC)	2 753 746	32.85	2 658	19	58	4	CP071100	[16]
PS/BAC/317/16 /W	Blood (PC)	2 665 983	32.93	2 392	19	58	4	CP071104	[17]
HOU1444-VR	Blood^a*^	2 798 705	32.79	2 836	19	59	4	CP012593	[18]
08–028	Blood^a*^	2 896 902	32.89	2 780	16	57	4	CP045435	[19]
PNID0137	Blood^a*^	2 914 236	32.50	2 847	19	58	4	CP071594	NCBI database
pt232	Blood^a*^	2 938 322	32.92	3 028	19	58	4	CP049991	NCBI database
GY8	Blood^a*^	2 788 282	32.88	2 698	19	58	4	CP092825	NCBI database
ER03864.3	Blood^a*^	2 873 608	32.79	2 965	19	59	4	CP030575	NCBI database
ER03910.3	Blood^a*^	2 830 468	32.90	2 877	19	59	4	CP030566	NCBI database
ER04013.3	Blood^a*^	2 840 211	32.99	3 010	19	59	4	CP030459	NCBI database
Pt194	Blood^a*^	2 840 208	32.83	2 904	16	58	4	CP049547	NCBI database
Pt299	Blood^a*^	2 795 175	32.85	2 711	19	59	4	CP049374	NCBI database
ATCC 25923	Non-blood^b^	2 778 854	32.88	2 697	19	58	4	CP009361	[20]
SG511 Berlin	Non-blood^b^	2 739 564	32.86	2 619	16	58	4	CP076660	[21]
MRSA252	Non-blood^b*^	2 902 619	32.90	2 803	16	59	4	NC_002952	[22]
NCTC13395	Non-blood^b^	2 766 239	32.91	2 647	19	58	4	LS483316	NCBI database
200	Non-blood (skin)^*^	2 719 166	33.00	2 569	19	59	4	CP077924	[23]
Toyko12480	Non-blood (vomit)^*^	2 869 819	32.85	2 793	19	60	4	AP019712	[24]
USA300_SUR15	Non-blood (abscess)^*^	2 919 910	32.79	2 957	19	61	4	CP014415	[25]
MW2	Non-blood (nose)^*^	2 820 462	32.83	2 706	19	58	4	NC_003923	[26]
CC5	Non-blood (tracheal)^*^	2 745 711	32.90	2 965	19	61	4	CP021105	[27]
USA300_2014 .C02	Non-blood (nose)^*^	2 864 345	32.79	2 864	19	59	4	CP012120	[28]

PC; platelet concentrate isolate, a; isolate from unspecified blood origin, b; isolate from unspecified non-blood samples, and *; involved in infection.

### Bacterial culture and DNA extraction

*S. aureus* isolates were streaked on Tryptic Soy Agar (Sigma) plates and grown overnight at 35 °C. Cells were collected in DNA/RNA Shield and DNA was extracted using the Zymo Quick-DNA HMW Magbead kit (Cedarlane) with RNaseA treatment and overnight 56 °C cell lysis (Lysozyme, Lysostaphin, Mutanolysin) as per the manufacturer (Zymo Research Inc). DNA was further purified using 0.8 x AMPureXP (Beckman-Coulter) and eluted in 10 mM Tris-Cl (pH8).

### Whole genome sequencing and *de novo* assembly

*S. aureus* strain ATR-20003 (ATR) was sequenced on a next-generation Miseq Illumina platform at the Bureau of Microbial Hazards, Health Canada (Ottawa, ON, Canada). Briefly, paired-end Illumina whole-genome shotgun (WGS) sequencing was performed using Nextera XT DNA libraries run on a MiSeq instrument (v3 chemistry, 2×300 bp reads) according to the manufacturer instructions (Illumina). Nanopore WGS sequencing libraries were constructed using the rapid barcoding sequencing kit (SQK-RBK004) and run using a FLO-MIN106 flow cell (R9.4) and a 1D MinION system (Oxford Nanopore Technologies).

The raw Illumina reads were processed using FastP (v0.20.1) [53] to remove adapter and barcode sequences, correct mismatched bases in overlaps, and filter low-quality reads (Q<20). Long-read signal processing, base calling, demultiplexing, and adapter trimming were performed using Guppy GPU v5.0.16+b9fcd7b and reads of <1 kb were removed using Filtlong v0.2.0 (https://github.com/rrwick/Filtlong). A *de novo* hybrid assembly was generated using Unicycler (v 0.4.8) using default settings [54]. Assemblies were annotated with PGAP v 2021-01-11. build5132 (best placed reference protein set, GeneMarkS-2+) (github.com/ncbi/pgap) and analysed with QUAST v 5.0.2 (github.com/ablab/quast).

### Genome comparison and phylogenetic analyses

The genomes of the *S. aureus* isolates from PCs were aligned and visualized using progressive Mauve (20 150 213 build 0) [55]. Phylogenetic analysis was performed using the closed genomes of PCs isolates against complete genomes of the 20 blood and non-blood *S. aureus* strains retrieved from GenBank. Core-genomes of all these strains were aligned using Parsnp (v. 1.2) [56], with *S. aureus* NCTC13395 as the reference strain. Maximum likelihood phylogenetic trees based on the Tamura-Nei model were constructed with mega (v10.1.8) [57], using the aligned sequences and 1000 bootstrap replicates. *S. aureus* strains were typed using PubMLST (https://pubmlst.org; accessed 13 May 2024 [58]. Isolates that did not have a defined sequence type (ST) were submitted to PubMLST for ST assignment [59]. Clonal complexes were identified with PHYLOViZ v2.0 [58], using the goeBURST algorithm.

### Virulence and AMR genes identification

To identify the virulence and AMR genes encoded in the genomes, the Virulence Factor Database (VFDB 2022) VFanalyzer9 (VFDB: Virulence Factors of Bacterial Pathogens ([mgc.ac.cn)] [accessed on 24 August 2022]) [60], and the Comprehensive Antibiotic Resistance Database (CARD-RGI) (The Comprehensive Antibiotic Resistance Database (mcmaster.ca) (accessed on 30 August 2022) [61], were respectively used under default parameters to curate the genes. The list of virulence and AMR gene accession numbers generated by VFBD and CARD-RGI runs were further verified by alignments with the whole genome sequences using BLASTn (v.2.12.0+) under default parameters and -perc_identity 70 [62,63]. The outputs of the blast analysis were manually verified and visualized using iTol (v6) [64]. T7SS virulence gene clusters were compared among PC isolates using strain USA300_SUR15 as reference. Multiple alignments of the *essC* nucleotide sequence using MultAlin (Multalin interface page (inra.fr) (accessed on 20 May 2023) [65], and conserved protein domains detected using the NCBI CDD search tool (NCBI Conserved Domain Search (nih.gov) (accessed on 27 May 2023) [66], were carried out on the strains. Genomic DNA extraction from *S. aureus* cells was performed using the GenElute bacterial genomic DNA kit per manufacturer instructions (Sigma-Aldrich, St. Louis, MO, USA). Core genes (*esxA*, *esaA*, *essA*, *esaB*, *essB* and *essC*) of T7SS machinery were PCR-amplified from the genomic DNA of *S. aureus* PC strains using gene-specific primers and OneTaq DNA polymerase kit procedure (NEB, Ipswich, MA, USA), with strains ATR-20003 as PC positive control and ATCC 25923 as non-blood positive control. The PCR was run under the following optimized conditions: 3 mins initial denaturation at 95 °C; 25 cycles of denaturation (30 s at 95 °C), annealing (1 min at 58 °C), and extension (1 min at 68 °C); a final extension at 68 °C for 5 mins [67]. The primer sets and expected amplicon sizes are listed in Table S1.

### Detection and comparison of MGEs

A genome-wide scan for mobile genetic elements (MGEs) was performed on the complete genomes of PC isolates via mobileOG-db (*beatrix-1.6*) in PROKSEE tool (Proksee - Genome Analysis [accessed 14 April 2023]), with default settings [68]. Predicted genomic islands were confirmed by running the genomes on IslandViewer4 software [69] and compared with the closest phylogenetically related blood and non-blood strains via IslandCompare tool [70]. The MGEs were identified as genomic islands (νSa), phages (ɸSaint), transposons (Tn55), and SCC elements. PHASTER (v4.3X) (accessed 17 May 2023) [71] was further used to identify the different bacteriophages with default parameters. The sizes of the detected islands and phages were manually corrected using publicly available νSa and phage sequences of reference strain (MRSA252) available at NCBI. The identified vSaβ were classified based on the nomenclature proposed by previous reports [50,51]. All detected MGEs were visually inspected closely for virulence determinants and AMR genes. Shared MGEs hosting virulence and AMR genes were compared among the PC isolates using *S. aureus* strain MRSA252 as a reference.

## Results

### Genome content and chromosome architecture

**Fig. 1.**
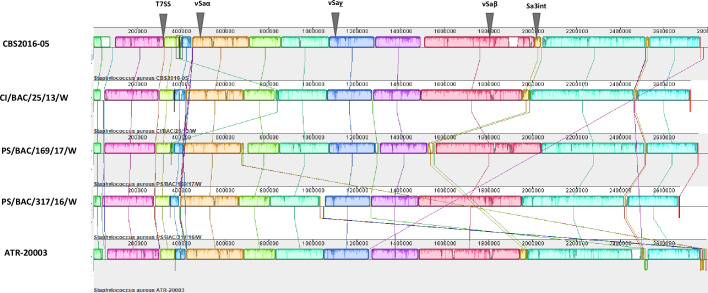
Multiple genome alignment of *S. aureus* strains from contaminated platelet concentrates using Mauve algorithm. Homologous DNA regions are shown as local colinear blocks of the identical colour containing the similarity profile of the genome sequence. The genome position is shown along the top of the alignments (bp) and isolate CBS2016-05 was used as the reference strain. Locations of genomic islands, prophage Sa3int and type VII secretion system (T7SS) gene cluster are indicated with grey triangles.

The main genome characteristics of *S. aureus* ATR-20003, reported in this study and other strains isolated from blood sources (including PCs) and non-blood samples are shown in Table 1. The chromosome size of PC isolates was between 2 665 983 bp in PS/BAC/317/16 /W and 2 766 936 bp in CBS2016-05, well within the range of reported genome size for *S. aureus* (2.5 Mbp to 2.9 Mbp). The sequences contained %GC ratios of 32.79 to 33.00, 19 tRNAs, four non-coding RNAs, and between 2392 to 2740, and 58 to 60 protein-coding and rRNA genes respectively. Multiple genome alignments revealed similar chromosome architectures. A total of eleven local co-linear blocks were obtained with syntenic breaks consistent with genome rearrangement events, mostly associated with MGEs profiles (Fig. 1). Inversion of insertional sequences was observed in strains ATR and PS/169 (ATR; 100 bp, 309 bp, 12 684 bp and 24 652 bp and PS/169; 167 bp and 1 794 bp). The approximately 24.65 kb inversion is located downstream in ATR (nucleotide position 2 764 273–2 788 925 bp), carrying MGE-like features, including an intact Tn55 transposon. Additionally, a rearrangement event involving insertion of a 11 783 to 13 354 bp fragment containing genomic features consistent with a prophage was detected within the genomes of CBS (nucleotide position 390 165–403 519 bp), CI/25 (nucleotide position 830 821–842 604 bp), and PS/169 (nucleotide position 353 364–365 212 bp). Two co-linear blocks located adjacent to each other of size range from 43 519 to 45 137 bp were present in all the analysed PC isolates but lacked the homolog sequence in strain PS/317 (Fig. 1). Examination of this region revealed the presence of genomic features consistent with ɸSa3int prophage integrated in the same genomic loci in the studied isolates.

### Phylogenetic relationship between blood and non-blood isolates

**Fig. 2.**
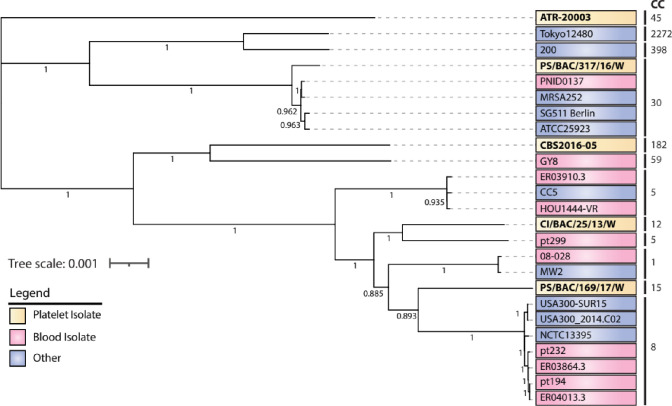
Phylogenetic relationship between *S. aureus* strains isolated from platelet concentrate, blood and non-blood (other) samples. A multi-alignment of the core-genomes was generated using Parsnp and Maximum Likelihood tree constructed using MEGA (v11.0.10) with 1000 bootstrap replicates. The scale bar represents the phylogenetic distance expressed as nucleotide substitutions per site. Bootstrap values are shown at each node. Clonal complexes (CC) were identified using PHYLOViZ. Reference sequences were obtained from GenBank with accession numbers shown.

Despite their variable clinical origins (blood-PCs and non-blood; skin, nose, trachea, vomit etc.), phylogenetic analysis of the representative core genomes revealed disparities between *S. aureus* population from blood and other sources (Fig. 2). Notably, the isolates from PC origin did not cluster with one another but belonged to different phylogenetic groups and clonal clusters (Fig. 2). The closest matches to the PC strains CBS2016-05, CI/BAC/25/13 /W and PS/BAC/317/16 /W were strains isolated from blood, being GY8, pt299 and PNID0137, respectively.

### Antimicrobial resistance genes

**Fig. 3.**
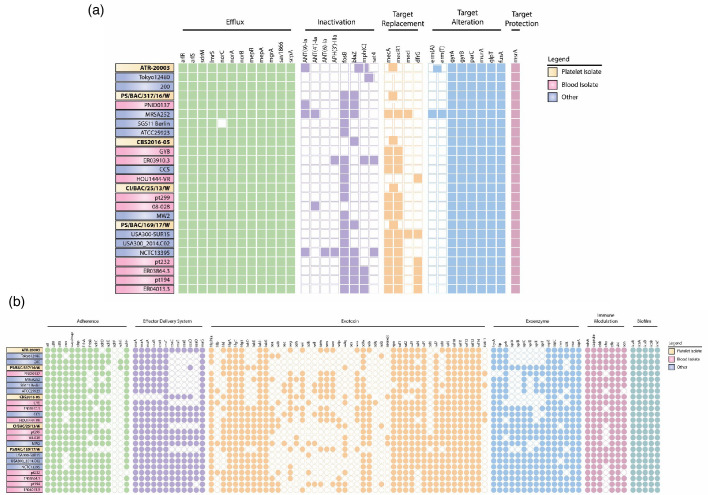
(a) Antibiotic resistant genes and associated antimicrobial resistance (AMR) mechanism in *S. aureus* strains isolated from platelet concentrates, blood and non-blood (other) using CARD-RGI database. Shaded squares of identical colour represent gene homologs across the different *S. aureus* isolates, while the white unshaded squares depict absence of the genes. (b) Virulence genes and corresponding virulence factor categories detected in *S. aureus* strains isolated from platelet concentrates (bold), blood and non-blood (other) using VFDB-VFanalyzer9 database. The shaded circles of identical colour represent genes associated with a specific virulence factor encoded by the different *S. aureus* isolates. The white unshaded circles depict absence of the genes.

The AMR profiles of the *S. aureus* genomes analysed revealed a variety of AMR genes, with each genome hosting a minimum of one gene coding for an efflux pump, target alteration, inactivation, protection, and replacement resistance mechanisms (Fig. 3a). Genes related to multidrug resistance, fluoroquinolones, penam, cephalosporin (*parC parE, gyrA, gyrB, norA, norB*), SMR (*sepA*), ABC efflux (*sav1866*) and MATE (multidrug and toxic compound extrusion) efflux (*mepR, mepA*) were present in all strains. Likewise, genes conferring resistance to methicillin (*mecA*), fosfomycin (*glpT, murA), fusidic (fusA*), erythromycin and streptogramin B antibiotics (*msrA*) were equally detected. Additionally, the multi-drug resistance genes, *arlR, arlS, mgrA, sdrM, norC* and *lmrs* were present in most PC isolates (72–88%) (Fig. 3a). This was proceeded by fosfomycin resistance gene *fosB* (19/25; 76%, including three PC isolates), then by mecR1 (14/25; 56%), and *blaZ* conferring penicillin resistance (10/25; 40%, present four PC strains). The resistance profiles were lowest for aminoglycosides (*ant(9)-Ia, ant(4`)-Ia, ant(6)-Ia*, *aph(3’)-IIIa*), *ermA, mphC, dfrG* and *sat4* genes (8–24%), with *mphC* as the least encoded gene *mphC*. Among PC isolates, only ATR harboured *ant(9)-Ia, ant(4`)-Ia* and *ermA*.

### Virulence assessment

The virulome of the PC isolates was similar to those of the blood and non-blood *S. aureus* genomes analysed, harbouring a plethora of virulence genes responsible for bacterial adherence, immune modulation, biofilm formation, type VII secretion system (T7SS), as well as exoenzymes and exotoxins (Fig. 3b). The most conserved virulence gene clusters were capsular biosynthesis (capsule) and intercellular adhesins *ica*-operon (*icaABCD, icaR*), while those encoding Ser-Asp rich genes (*sdr*) and staphylococcal enterotoxins (SEs) were the most varied subgroups. Gene clusters harbouring exoenzymes, such as cysteine protease (*sspABC*), and serine protease-like proteins (*splABCDEF*), were present in most isolates (60–100%). Enterotoxin gene cluster (egc) carrying *seg, sei, sem, sen, seo* and *seu* or *yent1* were identified in ten isolates (10/25; 40%), equally distributed between blood and non-blood strains. All strains had the complete *hlgABC* hemolysin gene set and either *lukED, lukHG*, or both of the bi-component leukocidin pore*-*forming toxins. In addition, the following virulence genes were conserved in all isolates; fibrinogen-binding (*clfA, clfB*, *efb, eap*, *fnbA, sdrC*), immune evasion (*hysA, adsA, ebh, ebpS, sbi, scn, spn*), superantigen-like toxins (*ssl1, ssl5, ssl9-ssl14*) and staphylococcal enterotoxin (*selx*) (Fig. 3b). The genes, *fnbB, coa, sdrD, sdrE, sdrG, sdrH,* and *coa* were relatively present (8–96%), with *sdrG* being the least frequent.

### Missing core genes in the type VII secretion system

**Fig. 4.**
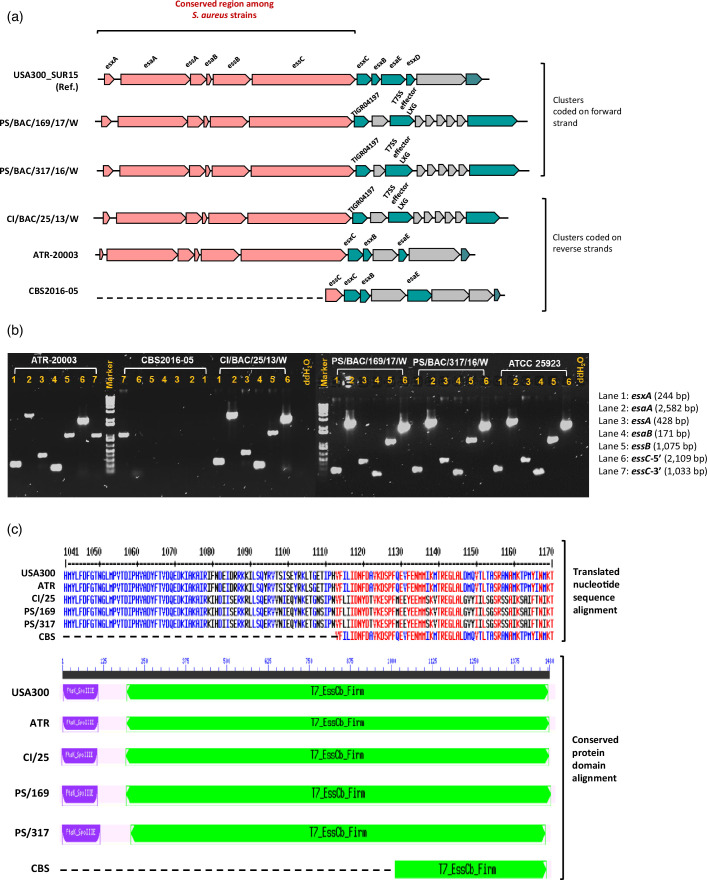
(a) Comparison of type VII secretory system (T7SS) gene cluster in *S. aureus* isolated from PCs. Alignment of T7SS gene cluster with strain USA300_SUR15 as reference. The upstream region of the cluster containing six genes (coral colour arrows) is highly conserved among *S. aureus* strains as shown above. Turquoise arrows indicate other known or predicted T7SS genes, and grey arrows represent other genes within the cluster. The broken line depicts the missing T7SS genes in CBS2016-05. (b) PCR confirmation of missing T7SS genes in *S. aureus* CBS2016-05 in comparison to other PC isolates. Genomic DNA was amplified using gene specific primers for the six core genes. Strain ATR-20003, a PC isolate that encoded the complete T7SS, having the full-length of essC according to bioinformatic analyses, was used as a positive control. A non-PC isolate, ATCC 25923 was also included as control for comparison with the PC strains. Platelet concentrate isolates: ATR-20003, CI/BAC/25/13/W, PS/BAC/169/17/W, PS/BAC/317/16/W and CBS2016-05. Non-PC positive control: ATCC 25923, and non-template control: ddH2O. (c) Multiple sequence alignment of EssC in *S. aureus* strains from PC origin, using NCBI CDD search tool. Below is an alignment of predicted conserved domains of EssC protein. The broken lines indicate absence of consensus sequence. USA300; USA300_SUR15, ATR; ATR-20003, CI/25; CI/BAC/25/13/W, PS/169; PS/BAC/169/17/W, PS/317; PS/BAC/317/16/W and CBS; CBS2016-05.

The T7SS gene cluster of the *S. aureus* effector delivery system was identified in all 25 genomes with *esaG* and *esaD* being the most and least abundant genes, respectively (Fig. 3b). While the complete T7SS gene cluster (size between15.5 kb and 19.8 kb) carrying core genes conserved across *S. aureus* strains was observed in four PC isolates (location indicated in Fig. 1), but not in strain CBS. The isolate had a reduced T7SS cluster of only 5.7 kb and was missing five of the six core genes (*esxA, esaA, essA, esaB* and *essB*). Only a truncated downstream fragment of *essC* (1 115 bp)*,* lacking approximately 3 336 bp (75% of the gene’s full-length) in the 5′ end, was present in CBS (Fig. 4a). This result was confirmed by PCR (Fig. 4b). *In silico* analyses revealed that the full-length *essC* encodes for a protein with two domains (FtzK_SpoI and T7_EssCb_Firm). While gene regions coding for both domains were observed in the tested strains, *essC* in strain CBS lacked the first domain and had only two-thirds of T7_EssCb_Firm domain, encoding for about a quarter of the full-length protein (Fig. 4c).

### Mobile genetic elements and rearrangements

**Fig. 5.**
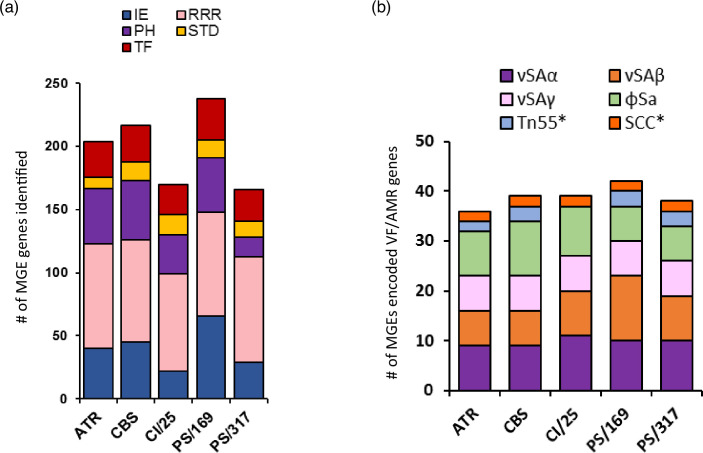
MGE features encoded by *S. aureus* isolated from PCs: (a) Number of genes associated with MGEs as predicted by mobileOG-db (beatrix-1.6). IE; integration/excision, RRR; replication/recombination/repair, PH; phage, STD; stability/transfer/defense, TF; transfer. (b) Number of virulence and antibiotic resistance genes detected in MGEs hosted by the isolates detected by IslandViewer4. The asterisks indicate MGEs encoding antibiotic resistant genes. ATR; ATR-20003, CI/25; CI/BAC/25/13/W, PS/169; PS/BAC/169/17/W, PS/317; PS/BAC/317/16/W and CBS; CBS2016-05.

**Fig. 6.**
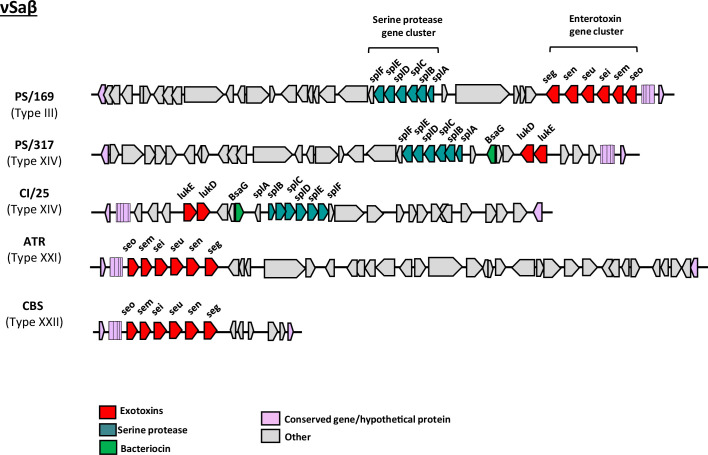
Comparison of νSaβ pathogenetic island organization and features across *S. aureus* isolated from PC samples. The genomic islands were identified using IslandViewer4. The different νSaβ types carry virulence determinants:(enterotoxins, leukocindins) and serine protease genes. ATR; ATR-20003, CI/25; CI/BAC/25/13/W, PS/169; PS/BAC/169/17/W, PS/317; PS/BAC/317/16/W and CBS; CBS2016-05.

**Fig. 7.**
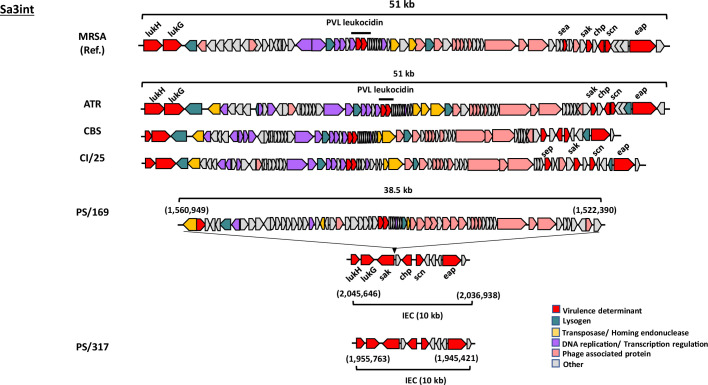
Structural organization of Sa3int phages in *S. aureus* isolates from platelet concentrates. PHASTER v4.3x tool was employed for phage identification. Phage size, gene orientation (arrows), and assigned functions are shown. MRSA252 was used as the reference strain. IEC; immune evasion cluster. MRSA:MRSA252, ATR; ATR-20003, CI/25; CI/BAC/25/13/W, PS/169; PS/BAC/169/17/W, PS/317; PS/BAC/317/16/W and CBS; CBS2016-05.

The analysed PC isolates revealed MGEs and genes associated with integration/excision (IE), replication/recombination/repair (RRR), phage (PH), stability/transfer/defence (STD) and transfer (TF) (Fig. 5a). Based on analyses of the complete genomes using mobileOG-db, genes associated with MGEs were identified [PS/169 (238), ATR (204), CBS (217), CI/25 (170) and PS/317 (166)]. RRR was the most abundant (34.5–50.6%), followed by IE or PH (9.0–27.7%), and then TF (13.4–15.1%). Detailed investigation of representative MGEs identified distinct νSa (νSaα, νSaβ, νSaγ), prophage (ɸSa3int) and transposon (Tn552) in the genomes of PCs isolates (Table 2, Fig. S1, available in the online version of this article), carrying virulence and/or AMR genes (Fig. 5b). The most conserved νSa was νSaγ that contained the immune evasion genes (*ecb*, *efb*, *scn*) and exotoxins (*hla, ssl*12-14) (Table 2). Based on νSaα classification [50,51], strains CBS, PS/169 and PS/316 had type I, while ATR and CI/25 contained type II, with all encoding exotoxins or the superantigen gene cluster (ssl1-5, 7–11) and the complement inhibitor (spn) (Table 2). More varied profiles were observed for νSaβ in PC isolates. Strains CI/25 and PS/317 harboured type XIV carrying serine protease gene cluster (*splA-F*) and the bi-component leukocidin toxins (*lukED*) (Fig. 6). ATR (type XXI), CBS (type XXII) and PS/169 (type III) had distinct νSaβ types, but hosted the enterotoxin gene cluster (*seg, sen, seu, sei, sem, seo*). Transposon Tn552 containing the β-lactamase gene locus, *blaZ, blaR1* and *blaI,* was detected in strains ATR, CBS, PS/169 and PS/317 (Table 2). The size of Sa3int phages in PC isolates ranged between 46.6 and 51.0 kb, similar to the reference strain MRSA252 (Fig. 7), except for PS/317 where its Sa3int was approximately 10.4 kb. The prophages are lysogenized at the same location. While three of the PC isolates had intact elements, PS/169 had about 10 kb of its sequence carrying immune evasion cluster (IEC) (*eap, scn, chp, sak*) and bi-component *lukHG,* translocated to another genomic location. This translocated sequence was similar to that observed in PS/317 (Fig. 7).

**Table 2.** Mobile genetic elements characteristics and corresponding virulence and antibiotic resistance genes encoded

MGEs	ATR-20003	CBS2016-06	CI/BAC/25/13 /W	PS/BAC/169/17 /W	PS/BAC/317/16 /W
νSaα size (bp) type genes	33 376 bp II ssl1, ssl2, ssl3, ssl4, ssl5, ssl9, ssl10, ssl11, spn	30 436 bp I ssl1, ssl4, ssl5, sssl7, ssl8, ssl9, ssl10, ssl11, spn	33 574 bp II ssl1, ssl2, ssl3, ssl4, ssl5, ssl9, ssl10, ssl11, spn	27 096 bp I ssl1, ssl2, ssl3, ssl4, ssl5, ssl9, ssl10, ssl11, spn	31 132 bp I ssl1, ssl2, ssl3, ssl4, ssl5, ssl9, ssl10, ssl11, spn
νSaβ size (bp) type genes	35 183 bp XXI seg, sen, seu, sei, sem, seo	9 779 bp XXII seg, sen, seu, sei, sem, seo	25 721 bp XIV splA, splB, splC, splD, splE, splF, lukED	31 583 bp III splA, splB, splC, splD, splE, splF, seg, sen, seu, sei, sem, seo	33 381 bp XIV splA, splB, splC, splD,splE, splF, lukED
νSaγ size(bp) genes	12 947 bp hla, ecb, efb, scn, ssl12, ssl13, ssl14	13 496 bp hla, ecb, efb, scn, ssl12, ssl13, ssl14	13 541 bp hla, ecb, efb, scn, ssl12, ssl13, ssl14	13 842 bp hla, ecb, efb, scn, ssl12, ssl13, ssl14	13 685 bp hla, ecb, efb, scn, ssl12, ssl13, ssl14
ɸSa3int size (bp) genes	51 031 bp PVL leukocins, lukHG, sak, chp, scn, eap	46 646 bp PVL leukocins, lukHG, sak, chp, scn, eap	48 182 bp PVL leukocins, lukHG, sak, chp, scn, sep, eap	49 147 bp PVL leukocins, lukHG, chp, scn, eap	10 392 bp sak, chp, scn, eap, eap
Tn552 size(bp) genes	3 936 bp BlaZ, blaR1, blaI	3 987 bp BlaZ, blaR1, blaI	na	4 470 bp BlaZ,blaR1, blaI	5 270 bp BlaZ, blaR1, blaI

## Discussion

The ability of *S. aureus* to infect, survive and persist in humans depends on the strain’s genomic content. Likewise, the outcome of transfusing PCs contaminated with *S. aureus* relies not only on the immune status of the patient receiving the PC unit, but also on the virulence factors produced by the bacterium. At the same time, the response to antibiotic treatment is based on the pathogen’s antibiotic-resistant phenotype. To understand the genome diversity of *S. aureus* isolated from PCs obtained either during routine PC screening or during investigations of transfusion reactions, we performed a detailed analysis of the genomes of five PC isolates in comparison to other *S. aureus* genomes from blood and non-blood sources, with focus on virulence and antimicrobial resistance.

Our investigation uncovered insightful information regarding genome rearrangements, virulence, and antibiotic resistance in *S. aureus* from PC origin. Multiple analyses of the complete genomes of PC isolates revealed syntenic breaks hosting MGEs and chromosomal rearrangements consistent with insertion, inversion, and translocation. Genome rearrangements are common phenomena in *S. aureus* genomes as reported for MRSA and ST239 strains [28,44]. The carriage of MGEs among *S. aureus* from PCs displayed a diverse structural repertoire regarding specific *S. aureus* genomic island (νSa) types and genomic loci of phages. Consistent with other *S. aureus* strains [50,72], the genomes of PC isolates had components of genomic islands, νSaα, νSaβ, νSaγ, ɸSa3int and Tn552. νSa and phages encoded adhesins, exoenzymes, exotoxins and immune evasion cascades, which facilitate pathogenicity by enhancing adherence, colonization, disease establishment, survival, and persistence [73,74]. PC recipients receiving platelet units contaminated with *S. aureus* usually suffer from septicemia caused by virulence factors expressed in the transfused PC during storage [75,76]. Moreover, genomes of PC isolates encoded multiple antimicrobial genes, including multidrug MATE efflux, ABC efflux and SMR families, which confer resistance to fluoroquinolones, penams, and cephalosporins, among others. Methicillin/penicillin resistance β-lactamase *mecA* and *blaZ,* common in *S. aureus* isolates [47,77], were also encoded, potentially complicating treatment options for *S. aureus* infections.

Structural arrangements and genomic loci of the incomplete ɸSa3int in PS/169 and PS/317 strains are signals of probable translocation and loss, respectively. The mobility and lifecycle of phages is well documented. During the lysogenic life cycle, a bacteriophage DNA is incorporated into the host bacterial chromosome where it replicates without destroying the host cell [78]. The ɸSa3int in PS/169 share similar types of holins, lysins and DNA packaging genes and harbour diverse DNA replication and virulence genes like other PC isolates and MRSA252. Considering the genetic content of ɸSa3int in PS/169 and the phage lysogenic lifestyle, a recent rearrangement event involving the relocation of its immune evasion cluster (IEC) to another locus within PS/169 genome may have occurred. Likewise, the loss of approximately 38.5 kb segment of the phage in PS/317, left behind the orphan virulence cluster (IEC ~10.0 kb) in the *S. aureus* host. Immune evasion proteins enable the bacteria to manipulate, avoid or inactivate host immune defenses, ensuring survival within the host [79]. The ɸSa3int IEC is likely important for the survival of *S. aureus* in harsh host environments like that of PCs, where bacteria are exposed to an array of immune factors such as antimicrobial peptides.

An unexpected finding was the loss of T7SS genes by *S. aureus* CBS2016-05. The prototype *S. aureus* T7SS contains six core proteins (EsxA, EsaA, EssA, EsaB, EssB and EssC) that interact with cytosolic chaperones, forming a channel through which proteins are secreted [39,40]. The T7SS in *S. aureus* CBS2016-05 lacked five of the six core genes with only a truncated *essC*. Typically, the missing genes are located immediately upstream of *essC,* as observed in other *S. aureus* strains, including those from PC origin. A recent study reported sequence conservation of T7SS core genes from *esxA-esaA-essA-esaB-essB* extending to the 5′-coding sequence of *essC*, and sequence difference at *essC* 3′ [80]. Potentially, a deletion rearrangement in CBS2016-05 excised the five core genes together with part of *essC*. The *S. aureus* T7SS is associated with a virulence phenotype by causing significant immune response, and also contributes to interbacterial competition [80]. Moreover, genes in T7SS cluster were reported to be upregulated in a mouse model of abscess formation, showing an increase in bacterial virulence and antagonism [81].

Within the T7SS machinery, substrates such as EsxA and EsxC cause significant immune response [82], and EsxA is known to be the core secreted component required for the secretion of all other substrates [83]. Additionally, the multi-domain ATPase EssC mediates substrate recognition and provides the energy required for substrate export, including transport of toxin proteins by the other T7SS entities. This would imply that loss of EssC and the core secretory EsxA, might not only affect the existence and activities of the other conserved genes, if present, but also destabilize the entire T7SS apparatus, thereby impacting its role in bacterial virulence and competition. Previous studies reported reduced infection and antagonism in T7SS mutant strains [39,81]. Perhaps, *S. aureus* CBS2016-05 does not require T7SS for interbacterial competition. Since T7SS exports several important proteins required for bacterial survival and persistence, *S. aureus* CBS2016-05 appears to have two options; (1) relying on the pseudo-T7SS, implying less virulence potential and impaired survival in harsh environments like that of PCs. Nontheless, this is not the case; despite having a defective T7SS machinery, *S. aureus* CBS2016-05 caused a septic transfusion reaction in a patient [12], had increased expression of virulence genes in PCs based on RNAseq studies [75], and secreted exotoxins in contaminated PCs [67]. Either the T7SS is not essential for *S. aureus* CBS2016-05 virulence or the bacterium over-expresses other virulence genes or compensates by recruiting other export mechanisms in response to a lack of a functional T7SS. (2) Alternatively, following the loss of T7SS core genes, CBS2016-05 employs T7SS proteins encoded outside the cluster and/or neutralizing immunity proteins. A study by Bowman and Palmer revealed the presence of multiple immunity protein-related genes in the genome of *Staphylococcus warneri* that lacked T7SS [78]. Interestingly, strain CBS2016-05 contains several genomic islands encoding a subset of immunity peptides (YezG, YeiH, FtsX-like proteins) that share similarity with the immunity proteins in *S. warneri*. Moreover, CBS2016-05 encodes potential T7SS substrate proteins proximal to the pseudo *essC* and outside T7SS cluster that might substantially play the role of T7SS in CBS2016-05.

## Conclusion

Community and hospital-acquired *S. aureus* infections involve strains with virulence and AMR genes acquired via MGEs that affect both bacterial genotype and phenotype. Comparative analyses of genomes of *S. aureus* isolated from PCs confirmed genome plasticity with variation in common Sa3int prophages that carry virulence genes in blood and non-blood strains. The phage Sa3int in two PC isolates, PS/BAC/169/17 /W and PS/BAC/317/16 /W are evidence of genome rearrangements. While the former had undergone translocation of its immune evasion cluster (IEC), the latter had lost part of the phage, leaving behind its IEC. Most importantly, T7SS in strain CBS2016-05 was unique as five of the core conserved genes were missing and the last gene was truncated. Since T7SS enhances *S. aureus* virulence by enabling protein transport and bacterial persistence, CBS2016-05 probably compensates for the role of T7SS by using other export mechanisms and/or employing alternative mechanisms such as neutralizing immunity proteins for effective bacterial virulence. This study unravels genome rearrangements in *S. aureus* isolated from PCs and reports the first *S. aureus* isolate that naturally lacks T7SS conserved core genes.

## supplementary material

10.1099/acmi.0.000820.v4 Uncited Fig. S1.

10.1099/acmi.0.000820.v4 Uncited Table S1.

## Abbreviations

AMR: antimicrobial resistance
IEC: immune evasion cluster
MDRSA: multidrug resistant *S. aureus*
MGEs: mobile genetic elements
PCs: platelet concentrates
SCC: staphylococcal, chromosome cassette
T7SS: type VII secretion system
WGS: whole genome shotgun
