# Supplementary material for: Comparative genome analyses of Staphylococcus aureus from platelet concentrates reveal rearrangements involving loss of type VII secretion genes
Source: Access Microbiol. 2024 Sep 13;6(9):000820.v4. doi: 10.1099/acmi.0.000820.v4 (PMC11652724; doi:10.1099/acmi.0.000820.v4)
Supplement: Uncited Fig. S1. [file acmi-6-00820-s001.pdf]

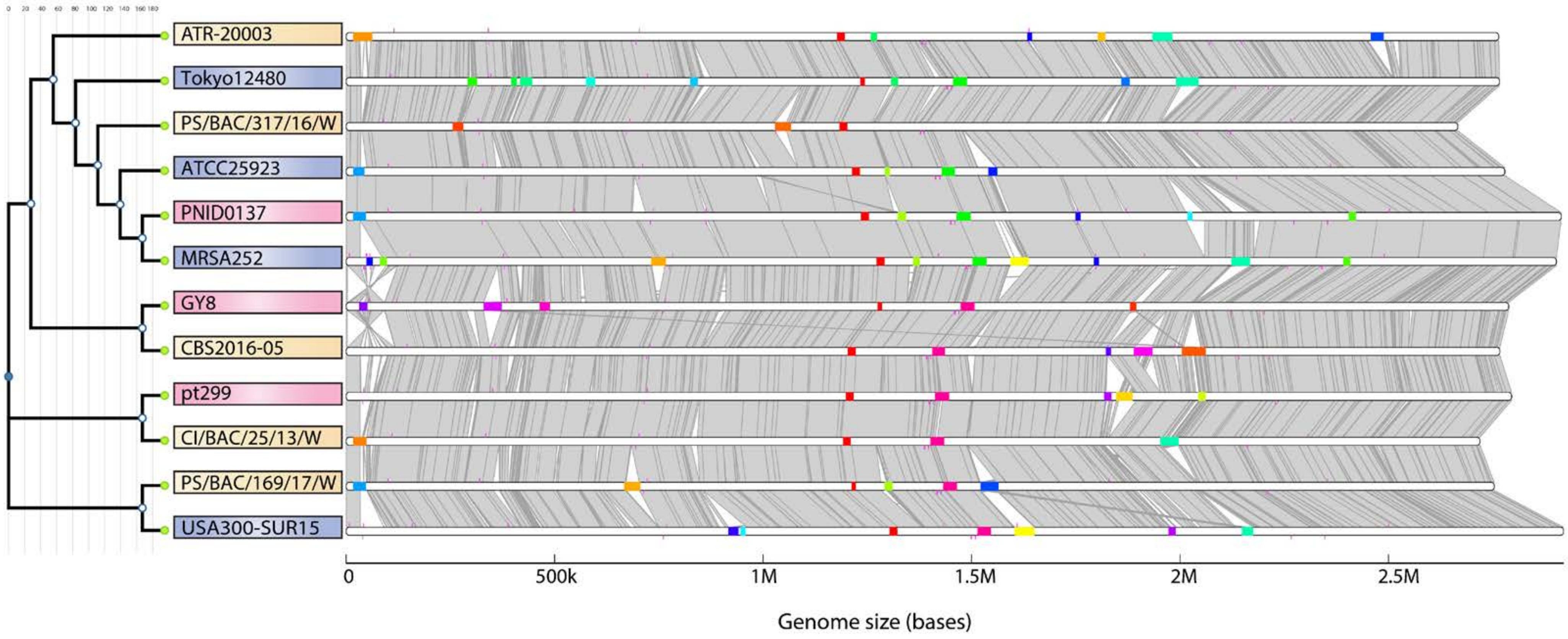

Supplementary Figure 1. Genomic islands detected across *S. aureus* isolates from platelet concentrates (pale yellow), blood (red) and non-blood (blue) using IslandCompare software. The phylogeny tree (left) indicates the relationship between the isolates analyzed. The linear white bars represent the genomes, and the grey shades indicate percent identity alignments between the genomes. The colored blocks on the linear genomes depict identified genomic islands.
