# Supplementary material for: Comparative genome analyses of Staphylococcus aureus from platelet concentrates reveal rearrangements involving loss of type VII secretion genes
Source: Access Microbiol. 2024 Sep 13;6(9):000820.v4. doi: 10.1099/acmi.0.000820.v4 (PMC11652724; doi:10.1099/acmi.0.000820.v4)
Supplement: Uncited Table S1. [file acmi-6-00820-s002.pdf]

Supplementary Table 1. Primer sets used for PCR amplification of core genes of *S. aureus* type VII secretion system. The primer were designed based on *S. aureus* ATR-20003 gene sequences.

Gene	Primer name	Oligonucleotide sequence (5` to 3`)	Size of amplicon (bp)
esxA	EsxA-F<br>EsxA-R	GGCAATGATTAAGATGAGTCCAGAG<br>GGCATCAGCAGTGCTATTCAA	244
esaA	EsaA-F<br>EsaA-R	ACGTTGCTGAGTCTGGTTT<br>TCCAGCCGCTTTCAAATTATTC	2,582
essA	EssA-F<br>EssA-R	TGTTGATGAATAGCGTGATTGC<br>TTTCGTTCGTCTCCCTTTATGA	428
esaB	EsaB-F<br>EsaB-R	GACTTAGCAGTACCAGCATATT<br>AATATCTCCATCAGCGATTTGA	171
essB	EssB-F<br>EssB-R	GCTGAATTAACTGAACTACGTGAC<br>GTTGCTTTAATTTCTCATCTTTCGC	1,075
essC	EssC-5'-F<br>EssC 5'-R	GGGATATGGCTCGAAGTACAAG<br>CGCACGACCTGGTAATGTAA	2,109
	EssC-3'-F<br>EssC 3'-R	AGATTCACCTTTCCAAGAAGTT<br>TGCGACCATATATGCTTCATTT	1,033
